# Sustainability of knowledge translation interventions: the evidence lacks evidence

**DOI:** 10.1186/s12916-023-03022-2

**Published:** 2023-08-25

**Authors:** Alexis Descatha, Marc Fadel

**Affiliations:** 1 Univ Angers, CHU Angers, Univ Rennes, Inserm, EHESP, Irset (Institut de Recherche en Santé, Environnement Et Travail) - UMR_S 1085, IRSET-ESTER, SFR ICAT, CAPTV CDC, Angers, France; 2 Department of Occupational Medicine, Epidemiology and Prevention, Donald and Barbara Zucker School of Medicine, Hofstra, Northwell USA

**Keywords:** Knowledge translational intervention, Systematic review, Prevention

## Background

Despite a large amount of research to for heath improvement in the medical field, it is unsure that all patient can have access to effective, sustainable, and cost-effective programs, services, and drugs [1]. To tackle this issue, in the last 20 years, there has been increasing attention on how to reduce the gap between evidence-practice and policy, which lead to the concept of knowledge translation [2]. Knowledge translation relates to “ensuring that stakeholders are aware of and use research evidence to inform their health and healthcare decision-making.” This definition includes a wide target audience for knowledge translation, including policy makers, professionals (practitioners), patients, family members, informal carers, researchers, and industry actors [1]. Beyond these aspects, the question of the sustainability of knowledge translation interventions became a growing issue since sustainability is a key to maintaining good health outcomes and confidence in these interventions in many public health domains [3].

## Main text

In this particular context, Veroniki et al. in their latest work “Efficacy of sustained knowledge translation interventions in chronic disease management in older adults: systematic review and meta-analysis of complex interventions” aimed to review published evidence of sustainable knowledge translation interventions and their efficacy, in one particular field: older patient with chronic disease [4].

The authors focused on 65 years and older adults since they represent the largest growing age group and many of them have serious medical chronic condition. The authors included all studies with interventions that lasted at least 12 months for patient aged 65 years and older with at least one chronic disease or their caregivers or any other actors of knowledge translation mentioned previously. The process engaged knowledge users, defined as “an individual who is likely to be able to use research results to inform their decisions about health policies, programs and practices (here 17 knowledge users including patient, funder, policymaker)” and involved integrated knowledge translation [2]. They used a complex but rigorous taxonomy from the Cochrane Effective Practice and Organisation of Care [5], and the behavior change technique taxonomy [6], and grouped them into three components: adherence, sustainability, and fidelity. A study-level data synthesis was performed using the mean difference for continuous outcomes (for instance Quality of Life) and odds ratio for dichotomous outcomes (for instance Quality of Care). Even though a large number of studies fulfilled the inclusion criteria (over 150), only 14 randomized control trial assessed sustainability, and among them, five also assessed adherence. None of them described the three components at the same time. The authors concluded that, overall, few trials evaluated the multiple dimensions of sustainability of any knowledge translation intervention. For those that reported on Quality of Life and/or Quality of Care, interventions were globally helpful, though improvement of other outcomes remains uncertain.

Some limitations have been considered by the authors like the number of study and their heterogeneity which led to difficulties for efficacy comparison, as it is complicated to consider very different trials at the same time if the disease or the outcome is different. The heterogeneity as the results might partly explain such results. The cost effectiveness analyses initially planned were also not realized for those reasons. One interesting aspect of the results is the decrease of included study since 2016, while there has been a growing number of research in all medical fields. Rather than a decrease of interest in such topic, a possible explanation would be that translational interventions have become obvious and not specifically highlighted anymore. The authors’ next step is to update the systematic review and conduct a network meta-analysis since the review has not been updated since 2020. Indeed, it became more and more important to anticipate that such high-quality reviews are time-consuming and expensive, and keeping them alive at least for duration of the work and submission process can be crucial. For high-priority question with many new papers everyday (“burning question”), a living systematic review might even be recommended with specific tools [7, 8].

## Conclusions

The authors concluded that, for the particular population of 65 and more with chronic condition, the overall efficacy of sustainability knowledge translation remains uncertain because it varies by effect modifiers, including intervention type, chronic disease number, comorbidities, and participants’ age. Even if the usefulness of sustainability knowledge translation is evident, the main conclusion that is highlighted is the need for more studies providing an operational and standardized measure of such intervention to be able to explore complex outcomes and could lead to robust conclusions regarding treatments and associated results. This applies to many public health fields and not only on treatments but also to the field of prevention where there is a need for sustainability knowledge translation [9, 10].

## Data Availability

Not applicable.
